# Progress or plateau? A 20-year systematic review of self-efficacy among TCFL teachers

**DOI:** 10.3389/fpsyg.2025.1678437

**Published:** 2025-10-28

**Authors:** Qian Shi, Jing Zhang, Wei Zhao

**Affiliations:** 1 School of Chinese Language and Literature, Yunnan University, Kunming, China; 2 School of Entrepreneurship, Zhejiang University of Finance and Economics Dongfang College, Haining, China

**Keywords:** teacher self-efficacy, teaching Chinese as a foreign language (TCFL), social cognitive theory, professional development, cross-cultural adaptation

## Abstract

This systematic review examined research on the self-efficacy of teachers of Chinese as a foreign language (TCFL) from 2004 to 2024. Guided by social cognitive theory and Bandura’s concept of self-efficacy, 15 empirical studies were synthesized following PRISMA guidelines, employing both qualitative and quantitative analyses. The review identified key factors influencing TCFL teacher self-efficacy, including personal, student, and environmental factors. It further showed that teacher self-efficacy predicts important outcomes such as technology use and integration, career development and retention, and emotional and psychological resources. The findings underscore the need for targeted professional development, supportive institutional policies, and cross-cultural adaptation resources, and they point to future research directions on emerging technologies and diverse teaching contexts.

## 1 Introduction

The concept of self-efficacy, defined as an individual’s belief in his or her ability to perform specific tasks successfully, originates from Bandura’s social cognitive theory. Since its introduction, self-efficacy has garnered extensive attention across disciplines and has become a cornerstone of psychological and educational research. In education, initial studies focused primarily on learners, exploring how self-efficacy influences learning behaviors and achievement. More recently, researchers have turned their attention to teachers, recognizing the significant role that their self-efficacy plays in shaping instructional quality (Liu et al., 2021). Empirical evidence has shown that teachers with higher self-efficacy positively affect students’ academic achievement and motivation (Cantrell et al., 2013; Mojavezi and Tamiz, 2012) and demonstrate more effective classroom management skills (Woolfolk and Hoy, 1990).

As one of the six official languages of the United Nations, China has gained strategic importance in global communication and diplomacy (Abu-Tineh et al., 2011). In the context of globalization, China’s economic growth has motivated an increasing number of people worldwide to learn Chinese language and culture for academic, professional, and cultural purposes. With this demand, China has invested heavily in promoting Mandarin Chinese internationally (Hartig, 2016; Xia et al., 2024). By 2021, Chinese-language education had expanded to more than 180 countries and regions, with over 70 countries integrating it into their national education systems (Bao et al., 2021). The number of learners surpassed 20 million globally (Jin and Cortazzi, 2006), creating sustained demand for qualified teachers of Chinese as a foreign language (TCFL; Lu and Geng, 2022; Sun et al., 2023).

The growing global demand has positioned TCFL teachers as key agents of cultural exchange and educational diplomacy. However, much of the teacher self-efficacy literature still concentrates on English language education (Han and Wang, 2021; Heng and Chu, 2023), and research on TCFL teacher self-efficacy remains limited. Several systematic reviews have synthesized teacher self-efficacy studies more broadly, for example, Klassen et al. (2011) highlighted the neglect of contextual factors in general teacher efficacy research, and Wyatt (2018, 2024) provided reviews of language teacher self-efficacy. Hoang (2018) also examined EFL teacher self-efficacy and noted the dominance of quantitative approaches and the limited exploration of links to student outcomes.

Against this background, a focused synthesis of TCFL teacher self-efficacy is needed to clarify research trends, methodologies, and thematic directions (Miller et al., 2017; Orakcı et al., 2023). Although scholarly interest in this topic has increased in recent years, no systematic review has yet mapped the field comprehensively. Therefore, this study aims to fill this gap by systematically reviewing the literature on TCFL teacher self-efficacy in accordance with PRISMA guidelines, thereby contributing to both research and practice in TCFL teacher development.

## 2 Literature review

### 2.1 Self-efficacy

Self-efficacy, a central concept in social cognitive theory, refers to individuals’ beliefs in their ability to execute actions required for achieving specific goals (Bandura, 1986, 1997). Unlike broader self-concepts such as self-esteem, self-efficacy is task-specific, focusing on confidence in performing particular tasks (Maroiu et al., 2016). These beliefs strongly influence choices, effort, and persistence: people who perceive challenges as exceeding their coping abilities tend to avoid them, whereas those with stronger efficacy beliefs engage more confidently (Bandura, 1977; Tschannen-Moran et al., 1998). Bandura identified four primary sources of self-efficacy: (1) enactive mastery experience—confidence built through successful performance; (2) vicarious experience—learning from observing others succeed; (3) verbal persuasion—encouragement and support from others; and (4) physiological and affective states—how stress and emotions shape efficacy beliefs (Bandura, 1977, 1986, 1994, 1997). Among these, mastery experiences are most influential, as success under cognitive challenge strengthens future confidence (Bandura, 1977, 1994; Tschannen-Moran and Hoy, 2007). Observing peers, particularly those perceived as similar, can also enhance efficacy through modeling (Hanif, 2010; Pajares, 1996). Verbal persuasion and emotional regulation help prevent declines caused by self-doubt or stress (Bandura, 1994; Hanif, 2010).

Self-efficacy is not an isolated psychological construct; rather, it is closely intertwined with other variables in the learning process (Wang D. et al., 2024). Foreign language anxiety has been identified as a key negative predictor of self-efficacy (*r* = −0.704), a relationship that remains stable across countries, educational levels, genders, and learning contexts (Zhou et al., 2023). Research also indicates that students’ writing self-efficacy is strongly associated with their use of self-regulatory strategies and can effectively predict their writing development (Sun and Wang, 2020). In addition, Wang et al. (2021)via latent profile analysis, identified three self-efficacy profiles among Chinese EFL undergraduates: low, moderate, and high. Students with high self-efficacy experienced more positive emotions (e.g., enjoyment, pride) and fewer negative emotions (e.g., anxiety, shame) and achieved higher scores in overall language proficiency as well as in listening and reading subtests. Although emotional differences were evident between the low- and moderate-level groups, no significant differences were observed in language performance. These findings highlight the central role of self-efficacy in shaping students’ emotional experiences and academic outcomes in language learning. As students’ beliefs and behaviors are often shaped by those of their teachers, understanding and fostering teacher self-efficacy are also crucial for enhancing instructional effectiveness (Shi et al., 2025; Sun and Wang, 2020; Wang D. et al., 2024).

### 2.2 Teacher self-efficacy

Teacher self-efficacy is defined as “teachers” who believe in their ability to organize and execute the courses of action required to produce given attainments” (Tschannen-Moran et al., 1998, p. 233). It is dynamic and cyclical: higher self-efficacy fosters greater effort and persistence, leading to improved performance, which in turn reinforces efficacy; conversely, low self-efficacy can perpetuate weaker outcomes (Bandura, 1997).

Teacher self-efficacy comprises three components: personal, organizational, and professional efficacy (Sela-Shayovitzhayovitz and Finkelstein, 2019; Wang et al., 2015). These three components interact collectively, influencing teachers’ performance and career development in their daily teaching practices (Sela-Shayovitzhayovitz and Finkelstein, 2019). Personal efficacy refers to teachers’ beliefs and attitudes regarding their roles. For example, teachers who positively view their profession are more motivated to innovate in the classroom. Organizational efficacy involves a support system within the school, such as teamwork and administrative support, which can improve or hinder teachers’ performance. Professional efficacy refers to teachers’ confidence in performing tasks effectively, such as lesson planning or managing classroom behavior. These components interact continuously and influence both daily teaching practices and long-term career development. For example, strong organizational support enhances both personal and professional efficacy, leading to a more motivated and resilient teacher capable of navigating classroom challenges effectively (Friedman and Kass, 2002). Recent empirical studies extend the domain of teacher self-efficacy beyond instructional performance. Wang X. et al. (2024) surveyed 375 Chinese middle school English teachers and found that teaching satisfaction and resilience mediate the relationship between self-efficacy and teachers’ well-being. This indicates that efficacy beliefs may influence psychological outcomes via affective and motivational pathways, not solely through classroom behaviors.

These findings suggest that a comprehensive review of TCFL teacher self-efficacy should not only examine teaching practices and student outcomes but also consider teacher psychological well-being and mediating mechanisms.

### 2.3 Existing systematic reviews and current goals

Several systematic reviews have investigated teacher self-efficacy across educational contexts, highlighting the central role of contextual factors. Klassen et al. (2011), in their first systematic review of teacher self-efficacy research, examined studies from 1998–2009 and emphasized the importance of considering local teaching environments. Building on this foundation, Wyatt (2018) conducted the first meta-analysis that specifically focused on language teacher self-efficacy, synthesizing research from 2005 to 2016. His review identified a growing use of qualitative and mixed-methods approaches and called for deeper context-sensitive investigations. Karas and Faez (2021) extended this line of inquiry by conducting a systematic review that reflected the continued expansion and diversification of language teacher self-efficacy studies. Similarly, Hoang (2018) focused exclusively on EFL teachers and analyzed studies from 2002 to 2017 and found a predominance of quantitative research, with little evidence linking self-efficacy to student learning outcomes. More recently, Wang X. et al. (2024) employed bibliometric analysis to examine 250 publications on language teacher self-efficacy from the Web of Science Core Collection (2003–2023), identifying publication trends, influential works, and key contributors. While offering a broad overview of the field, their study is limited by its exclusive reliance on a single database and the descriptive nature of bibliometric methods, which lack an in-depth content analysis.

In addition to language education, several reviews have explored teacher self-efficacy in other domains. Scarparolo and Subban (2021) reviewed the self-efficacy of differentiated instruction by former teachers from 2003 to 2018. Their review included four studies and revealed that self-efficacy is an area worthy of further research considering the link between teacher self-efficacy beliefs and their teaching. Through a 71-year systematic review, Wray et al. (2022) explored how teacher self-efficacy was measured and the factors influencing teacher self-efficacy in inclusive schools. It was found that teaching experience and the teaching environment influence self-efficacy. Knowledge of inclusive education policies increases teacher self-efficacy beliefs. There were also effects on confidence in teaching in inclusive classrooms, preservice teacher education, professional learning, and experiential engagement with disabled people. Gordon et al. (2023) explored the interaction between curriculum or assessment reform and teacher self-efficacy by analyzing 23 empirical studies to identify the factors that influence teacher self-efficacy during change, the support mechanisms needed to maintain high self-efficacy, and the support mechanisms needed to maintain high self-efficacy. This study reported that environmental determinants reduced teacher self-efficacy during the reform process. Professional learning to support high self-efficacy is necessary.

Given Bandura (1994) assertion that self-efficacy is context dependent, recent research has increasingly focused on TCFL teacher self-efficacy. The growing emphasis on TCFL self-efficacy, along with the expanding literature in this area, underscores the need for a systematic review. Accordingly, this study addressed the following research questions:

Q1: What are the research themes and key findings regarding TCFL teacher self-efficacy?
Q2: What are the methodological characteristics of TCFL teacher self-efficacy research studies?
Q3: What factors affect TCFL teacher self-efficacy?
Q4: What variables are predicted by TCFL teacher self-efficacy?

## 3 Methods

This systematic literature review was conducted in accordance with the PRISMA guidelines and a flowchart. The PRISMA guidelines comprise a 27-item checklist and three-stage flowchart delineating the requisite elements for transparency in a literature review (Page et al., 2021).

### 3.1 Literature search

A comprehensive search of the Web of Science, Scopus and ProQuest databases was conducted on 8 March 2024 to identify all papers published between January 2004 and that date. The following keywords were used for the search: “teacher (’s) (self-)efficacy” OR “teachers (’) (self-)efficacy” OR “teacher’s sense of efficacy” OR “teachers’ sense of efficacy” OR “teaching efficacy” AND “Chinese language teacher” OR “CFL” OR “teaching Chinese as a foreign language” OR “International Chinese teacher” OR “teaching Chinese as a second language” OR “CSL.” The search yielded 367 articles. In step two, duplicates were removed, leaving 334 articles.

### 3.2 Inclusion and exclusion criteria

**Figure 1:**
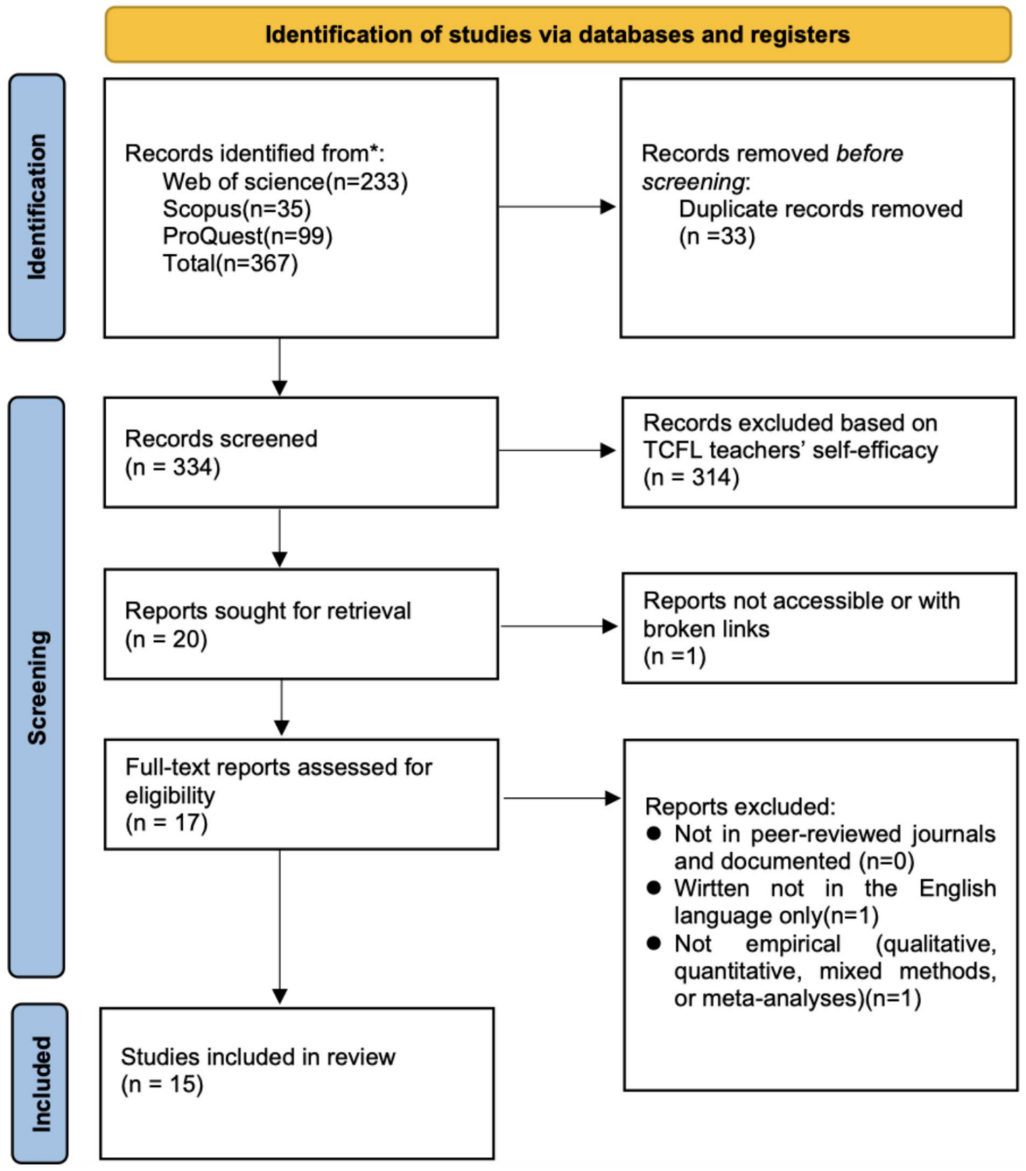
The review process [adapted from Tian et al., 2024]. While doctoral dissertation are not peer-reviewed journal articles, they were included in this review only when they demonstrated sufficient academic quality and relevance. Their inclusion also helps reduce potential publication bias, as they may present findings—especially null or negative results—that are less likely to be published in journal articles, thereby counteracting the ‘file drawer’ effect (Rosenthal, 1979; Wang and Devitt, 2022).

A Preferred Reporting Items for Systematic Reviews and Meta-Analyses (PRISMA; (Di Mascio et al., 2020)) statement flowchart (Figure 1) was constructed to clearly outline how the included studies were selected. The inclusion criteria were as follows:

- Studies related to TCFL teacher self-efficacy
- Studies published in peer-reviewed journals or doctoral dissertation written in English, provided that they meet the same quality standards in terms of methodological rigor, data completeness, and theoretical contribution
- Studies published from 2004–May 2024
- The study was empirical (qualitative, quantitative, mixed methods, or meta-analyses).
- The extracted data align with the current study’s focus and research questions.
- The full text was available

### 3.3 Data extraction

Following the initial search, 367 potentially eligible studies were identified: 233 from Web of Science, 35 from Scopus, and 99 from ProQuest. After removing duplicates, 334 studies remained. In line with the procedure adopted by Martins et al. (2022), the titles and abstracts of these studies were independently screened by two researchers according to the pre-established inclusion and exclusion criteria. When disagreements arose, the two reviewers discussed the cases to resolve inconsistencies; if consensus could not be reached, a third researcher was consulted. In all cases, consensus was ultimately achieved for the included studies, which helped ensure rigor and reduce potential subjectivity in the selection process.

This review excluded studies for the following reasons: (1) teacher self-efficacy was not examined as a variable, (2) TCFL teachers were not utilized as a sample, (3) it was not an empirical study, and (4) it was not written in English. Of the remaining 334 studies, 15 met the inclusion criteria. We conducted a thorough examination of these studies to form our final study, based on which we answered the questions posed in this study.

## 4 Results

Our final sample comprised 12 journal articles and three doctoral dissertations (see Table 1). This dataset enabled a thorough examination of TCFL teacher self-efficacy by incorporating a range of empirical perspectives.

**Table 1:** Data extracted from the included studies.

Author (s) (year)	Participants	Research directions	Research instruments and methods	Findings
Polin (2023)	47 US TCFL secondary teachers participated in the survey, and 8 teachers were interviewed.	Professional development (PD) and teacher self-efficacy.	**Instruments:** self-efficacy scale (Haley et al., 2013) and can-do statements were primarily based on the professional standards for K-12 Chinese language teachers (Lee et al., 2007). **Methods:** explanatory sequential mixed-methods design (Creswell and Plano Clark, 2018).	There is a significant relationship between teachers’ PD and their self-efficacy. However, self-reported PD support and PD needs were not statistically associated with self-efficacy. PD support, however, emerged as a significant predictor of self-efficacy.
Sun and Mei (2022)	331 TCFL pre-service teachers in China	Pre-service TCFL teachers’ intentions to use technology and examine the factors that influence their intentions, with self-efficacy being one of the influencing factors	**Instruments:** self-reported perceptions of perceived usefulness (Davis, 1989), attitudes toward use of technology (Edison and Geissler, 2003), technology self-efficacy (Compeau and Higgins, 1995; Hocevar et al., 2014), facilitating conditions (Lai, 2015; Teo, 2009), experience of technology use (Jeong and Kim, 2017; Lai et al., 2012), and intention to use technology (Armenteros et al., 2013; Davis, 1989). **Methods:** Structural equation modeling analysis using AMOS software.	Perceived usefulness, technology self-efficacy, and facilitating conditions positively influenced attitudes toward technology use. Moreover, perceived usefulness, attitudes toward technology use, and past technology use experiences positively affected intentions to use technology.
Liu and Sayer (2016)	2 focal teachers	Using the concepts of teacher efficacy and self-efficacy (Bandura, 1997), the beliefs and practices of 2 TCFL teachers working in a large Texas high school were explored. They identified four main factors that influence teacher efficacy.	**Instruments:** direct observations, participant observations, interviews, archival records, physical artifacts, documents. **Methods:** exploratory case study and fieldnote entries.	Self-efficacy among foreign language teachers is influenced by multiple factors, including school context, educational policies, types of foreign language programs, and opportunities for PD.
Bao et al. (2021)	1 frontline TCFL teacher from the Project	Teachers’ personal efficacy, managerial efficacy and professional efficacy	**Instruments:** written and spoken narratives. **Methods:** narrative inquiry and thematic analysis.	The self-efficacy of online Chinese language teachers is shaped by the interplay of external and internal factors.
Sun (2021)	3 L2 Chinese assistant teachers teaching beginner-level Mandarin to college students.	Factors influencing the motivation of TCFL assistants to participate in teaching and the critical impact of teaching self-efficacy on the career retention.	**Instruments:** semi-structured interview, drawing on the expectancy–value theory and the motivational FIT choice model. **Methods:** multiple-case-study.	Teaching assistants (TAs) maintained student-centered expectations regardless of background. Different values regarding Chinese language teaching impacted TAs’ teaching performance and career choices differently. Teaching self-efficacy critically, yet controversially, influenced TAs’ career retention, considering their prior specializations.
Sun et al. (2023)	3 novice TCFL teachers	Changes in the professional commitment of junior TCFL teachers in New Zealand and how teacher self-efficacy affects their professional commitment using an Integrated theoretical framework.	**Instruments:** interview and classroom observation data and instructional materials. **Methods:** case study approach: Through a combination of participant interviews, classroom observations and analysis of instructional materials.	Low or high teaching self-efficacy could hinder the professional commitment of junior teachers. Strong teaching self-efficacy combined with high outcome expectations significantly boosts professional commitment. Professional autonomy and social support can moderate the effect of teaching self-efficacy on professional commitment.
Lin and Zheng (2015)	19 foreign language teachers, including 7 teaching Chinese, 7 teaching Spanish, 3 teaching French, 1 teaching German, and 1 teaching Japanese	Teaching practices and teachers’ perceptions in online foreign language courses.	**Instruments:** instructional self-efficacy, Technology self-efficacy, Teaching practices and PD scales. **Methods:** mixed research method.	A range of instructional practices are commonly employed by online foreign language instructors, with maintaining academic integrity, effective communication, and student support being the most prevalent. Instructional practices can be grouped into content-related and non-content-related categories, with the former being less frequently utilized. Teachers’ instructional practices are influenced by teaching experience, instructional self-efficacy, and technology self-efficacy. Adjustments are necessary for teachers transitioning from face-to-face to online teaching, such as adapting to the new environment, altering teaching methods, and fostering effective communication with students.
Chan (2008)	273 pre-service and in-service teachers in Hong Kong, China, of whom 38 were Chinese language teachers	The role of emotional intelligence, self-efficacy and coping strategies among prospective and in-service teachers in Hong Kong.	**Instruments:** assessment tools for emotional intelligence and self-efficacy. **Methods:** quantitative research.	Personal and interpersonal emotional intelligence were significant predictors of positive coping strategies, whereas teacher self-efficacy did not independently predict positive coping strategies. Evidence suggests that teacher self-efficacy might interact with personal emotional intelligence in predicting positive coping strategies, especially for male teachers.
Chen and Yeung (2015)	Teacher researchers involved in the teaching of Chinese in schools in Western Sydney, Australia, who are Chinese university graduates and native speakers of the Chinese language	Factors influencing beginning teacher self-efficacy in TCFL in Australia.	**Instruments:**20 master’ s theses on teaching Chinese in Western Sydney Schools, Australia, completed by teacher fellows during the research-based teacher education program. **Methods:** qualitative research approach using coding and analysis of theses.	Three categories of factors influencing teacher self-efficacy were identified: teacher factors (including knowledge, skills, and experiences), student factors (such as student characteristics), and situational factors (like relationships with school administrators and parents). Teachers’ perceptions of their English proficiency were linked to self-efficacy, as was recognizing the benefits of being a bilingual teacher.
Bao et al. (2016)	The participant is the first author, a long-time English language learner and a TCSOL teacher in New Zealand	TCFL teachers’ perspectives and understanding of Communicative Language Teaching (CLT).	**Instruments:** first author’ s life history narrative, lesson plans, memos, field notes, and classroom recordings. **Methods:** narrative inquiry and thematic analysis.	Content-specific and self-efficacy beliefs are primarily shaped or reshaped by mastery/performance experiences, particularly those that are challenging. Positive social persuasion can be pivotal in strengthening self-efficacy beliefs after a traumatic experience, potentially leading to new mastery/performance experiences. Successful experiences enhance one’ s confidence in handling future obstacles.
Zhu and Li (2023)	2 TCFL teachers	Collective resilience of teachers in transnational higher education.	**Instruments:** policy papers, interviews and reflective journals. **Methods:** qualitative phenomenological approach.	A multi-layered contextual environment significantly contributes to building teachers’ resilience by promoting synergistic individual and institutional growth through collaborative efforts. Individual resilience influences institutional development, and the mechanisms of interaction between individual and contextual resources are highlighted. Collective resilience among language teachers is a multifaceted concept reflecting their response to contextual challenges through personal and collective growth and emphasizing the importance of individual resilience for institutional advancement.
Yang and Lou (2024)	In-service TCFL Teachers in China	Relationship between TCFL teacher self-efficacy and acceptance of technology in digital literacy	**Instruments:** TCFL Teachers’ Digital Literacy Self-Efficacy Questionnaire and Technology Acceptance Questionnaire. **Methods:** structural equation modeling.	There was a significant positive correlation between digital literacy self-efficacy and technology-related factors among TCFL teachers, and digital literacy influenced perceptions and willingness to use technology.
Liu (2020) PhD thesis	16 participants in the STARTALK summer residential PD	CFL teachers’ experiences developing technology skills and the types of support promoting technology integration in the language classroom, including the impact of TPACK self-efficacy on technology integration.	**Instruments:** documents, semi-structured interviews, audio-recorded Q/A sessions, audio-recorded counseling sessions and classroom observation field notes. **Methods:** multiphase mixed methods (Convergent Intervention Case Study, Q/A sessions support, participatory mentoring, and survey research).	TPACK self-efficacy and technology grit play an important role in TCFL teachers’ experiences of acquiring technology integration skills.
Polin (2021) PhD thesis	47 questionnaires were administered, and 8 individuals were selected for interviews.	NC secondary school teacher self-efficacy in applying professional standards and PD activities and needs.	**Instruments:** online survey on self-efficacy in applying professional standards and their PD activities and needs. **Methods:** explanatory sequential mixed methods research design(Creswell and Plano Clark, 2018).	Quantitative results indicated that teachers perceived benefits of PD activities were significantly and positively related to self-efficacy. While self-reported PD support was significantly associated with self-efficacy, PD needs showed no relation. Qualitatively, many teachers lacked a clear understanding of professional standards. They found the greatest benefit in gaining new perspectives and sharing with colleagues, though issues with PD content or organization limited benefits. Teachers’ most urgent PD needs were engaging students and differentiating instruction.
Liu (2012) PhD thesis	6 TCFL teachers	The relationship between teachers’ pedagogical beliefs and perceived efficacy in two cities in southern Texas.	**Instruments:** consisting of manifold methods of data collection (including direct and participant observation, interviews, artifact collection, and document review). **Methods:** muti-sited exploratory case study.	For novice and experienced teachers, consistent segregated work environments, prior learning and social experiences, and online resources formed the core of their teaching beliefs. Macro- and micro-situational factors were significantly associated with teachers’ beliefs, affecting their emotional states and expectations for professional and student achievement. These personal experiences and situational factors led to varied perceptions of personal and pedagogical efficacy, influencing views on curriculum design.

### 4.1 Research themes and key findings

An analysis of the included studies revealed five main research themes. (1) Professional development and needs: Several studies emphasized how professional development activities, opportunities, and perceived needs are closely tied to teachers’ self-efficacy (Liu and Sayer, 2016; Polin, 2021, 2023). (2) Technology use and integration: Research highlighted the role of technological self-efficacy in shaping teachers’ willingness and ability to integrate digital tools into their practice (Lin and Zheng, 2015; Liu, 2020; Sun and Mei, 2022; Yang and Lou, 2024). (3) Career development and professional commitment: Teacher self-efficacy was shown to influence career intentions, teaching motivation, and long-term professional engagement (Sun, 2021; Sun et al., 2023). (4) Contextual and institutional influences: Findings indicated that student-related factors, school environment, administrative support, and broader institutional conditions play an important role in shaping teachers’ efficacy beliefs (Bao et al., 2021; Chen and Yeung, 2015; Liu, 2012). (5) Resilience, emotional resources, and coping strategies: Studies also examined how self-efficacy interacts with emotional intelligence, social persuasion, and resilience, underscoring its contribution to teachers’ psychological resources and adaptability (Bao et al., 2016; Chan, 2008; Zhu and Li, 2023).

These themes demonstrate that TCFL teacher self-efficacy is a multifaceted construct shaped by personal, technological, professional, contextual, and emotional dimensions, with important implications for teacher development and retention.

### 4.2 Main methodological characteristics

The 15 studies reviewed presented diverse methodological characteristics in terms of their sample, design, and measures. The sample sizes varied widely, from single-participant case studies to surveys of 331 teachers, reflecting both in-depth qualitative investigations and broader quantitative analyses. In terms of research approach, qualitative studies were the most common (*n* = 8), followed by quantitative (*n* = 3) and mixed-methods studies (*n* = 4). The specific designs employed ranged from multiple case studies and narrative inquiries (for rich contextual insights) to cross-sectional surveys and a few longitudinal or intervention studies for temporal analysis. Most studies relied on purposeful sampling of participants (*n* = 11), with a smaller number using convenience samples (*n* = 4) to select teachers relevant to TCFL contexts. Data analysis techniques corresponded to the study types: thematic analysis was frequently used in qualitative studies (*n* = 4), whereas statistical methods, such as multiple regression (*n* = 3), structural equation modeling (*n* = 2), and correlation analysis (*n* = 2), were applied in quantitative research. Regarding the assessment of teacher self-efficacy, the majority of studies used teacher self-report measures—typically questionnaires (often adapted from established teacher efficacy scales) and/or interviews—to gage efficacy beliefs. One notable study (Chen and Yeung, 2015) took an unconventional approach by analyzing a set of Master’s theses to extract themes related to teacher self-efficacy influences rather than directly surveying teachers. The prevalence of qualitative and mixed-methods approaches in this body of work aligns with the field’s recognition of self-efficacy as a context-dependent complex construct.

Overall, the methodological variety observed (in terms of participant numbers, study designs, and data sources) highlights a comprehensive effort to understand TCFL teacher self-efficacy from multiple angles while also indicating the need for triangulation not only across methods but also across data sources to further strengthen the evidence base. The diversity in study designs and methodologies highlights the multifaceted nature of research on teacher self-efficacy, with purposeful sampling and prominent self-report measures. This indicates a trend toward a comprehensive understanding of the factors that influence teacher self-efficacy across different contexts and populations.

### 4.3 Factors influencing TCFL teacher self-efficacy

Across the reviewed studies, the factors influencing TCFL teacher self-efficacy can be broadly divided into internal and external factors. Internal factors mainly refer to personal factors, such as teachers’ language proficiency, pedagogical knowledge, teaching skills, prior experiences, and self-confidence. External factors include both student factors—for instance, learners’ motivation, classroom behavior, and teacher–student relationships—and environmental factors, such as administrative support, relationships with parents, class size, and resource availability. Student-related influences were particularly highlighted in Chen and Yeung (2015), who illustrated this categorization in their study of novice TCFL teachers in Australian schools, noting that learners’ motivation, classroom behavior, and teacher–student relationships significantly shape teachers’ efficacy beliefs. Similarly, Bao et al. (2021) demonstrated that in an online teaching context, personal (internal) confidence and mastery experiences, together with external supports such as institutional backing and a sense of belonging, strengthened teacher self-efficacy, whereas the absence of such supports weakened it.

Support from the surrounding environment has emerged as having a critical influence on teacher self-efficacy. Sun et al. (2023) adopted a case study approach focusing on professional commitment changes among three junior TCFL teachers who demonstrated increased self-efficacy, outcome expectations, professional autonomy, and social support. However, they reported that teaching in non-target-language settings presents unique challenges due to linguistic, social, professional, and cultural factors. Polin (2023) studied the self-efficacy and PD of TCFL teachers in North Carolina via a mixed-method approach and revealed that perceived PD benefits and support significantly influence self-efficacy. Regular critical reflection and active participation in the professional community are key to developing teacher self-efficacy. Liu and Sayer (2016) examined the role of self-efficacy in the teaching practices of TCFL teachers in the US, highlighting how self-efficacy influences their instructional adjustments amidst challenges and the impact of financial support and enrollment on self-efficacy beliefs.

In addition, a doctoral dissertation by Polin (2021) discussed the self-efficacy of Chinese second language (CSL) teachers in the application of professional standards. This study revealed a statistically significant correlation between participants perceived benefits of PD activities and self-efficacy. However, there was no statistically significant correlation between self-reported PD support and self-efficacy or between PD needs and self-efficacy. Perceived benefits of PD activities and actual PD support emerged as significant predictors of self-efficacy, while PD needs were not. Another doctoral dissertation explored the relationship between teachers’ instructional beliefs and their perceived efficacy. The study revealed that teachers’ perceptions of personal and instructional efficacy varied due to the influence of personal experiences and situational factors, which, in turn, affected their perceptions of curriculum design (Liu, 2012).

**Figure 2:**
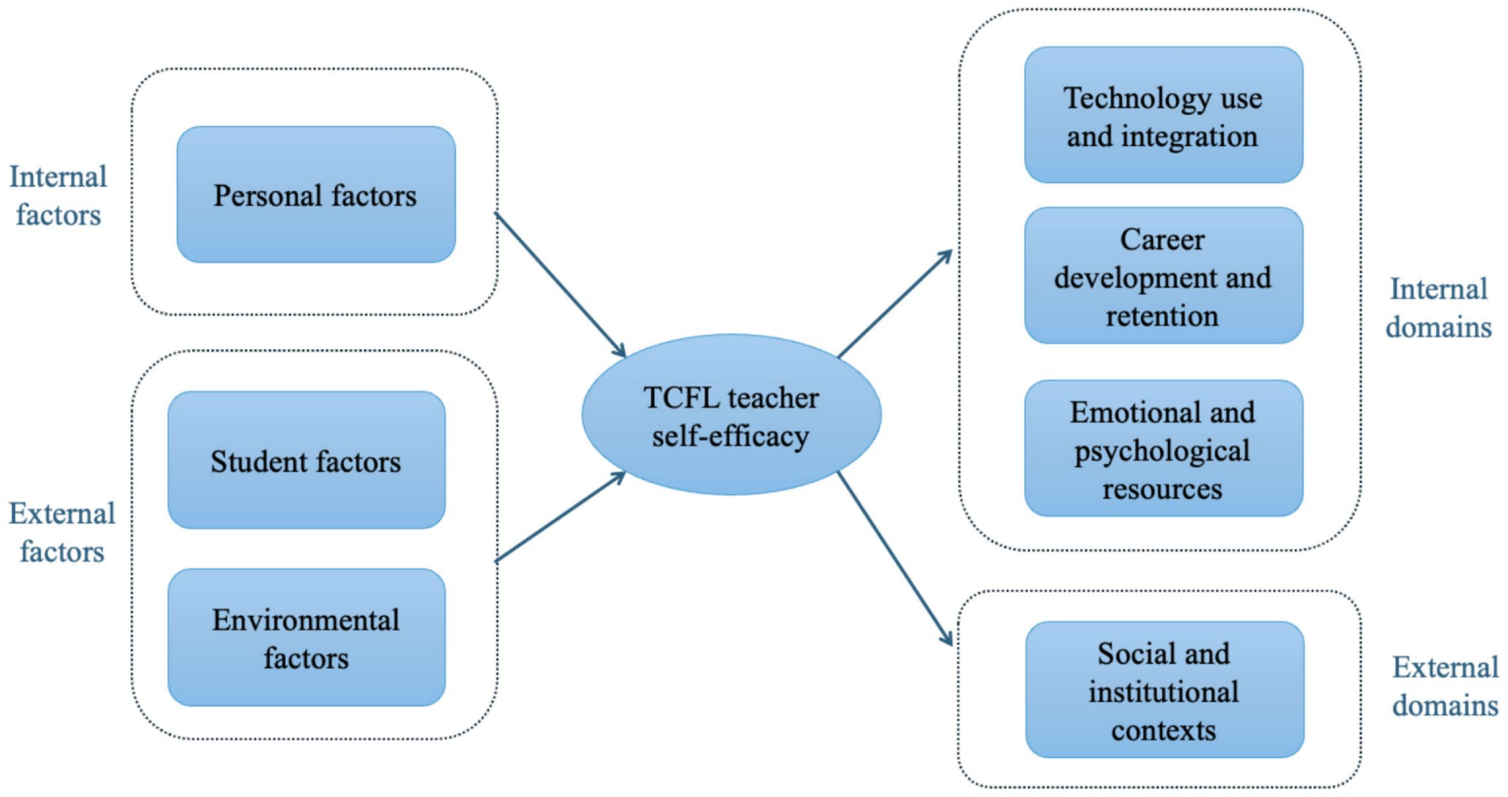
Variables related to TCFL teacher self-efficacy.

In summary, these studies collectively indicate that teacher self-efficacy is shaped by a complex interplay of personal, student, and environmental factors, with both internal beliefs and external support systems playing significant roles in fostering effective teaching practices. The findings highlight that PD, particularly when perceived as beneficial, significantly bolsters self-efficacy, although merely providing support for PD may not directly enhance it. Additionally, teachers’ instructional beliefs and perceptions of personal and instructional efficacy are influenced by their personal experiences and situational factors, impacting their approach to curriculum design. The evidence suggests that a multifaceted approach addressing these diverse influences is essential to effectively enhance teacher self-efficacy, especially within the specific context of TCFL. These relationships are summarized in Figure 2.

### 4.4 TCFL teacher self-efficacy and other variables

To clarify the predictive scope of TCFL teacher self-efficacy, the related variables can be grouped into internal domains, referring to variables closely tied to teachers’ own professional practices, career trajectories, and psychological resources, and external domains, which involve the broader institutional, social, and collective contexts in which teachers work.

In terms of internal domains, one prominent area is technology use and integration. Lin and Zheng (2015) found that both teaching and technological self-efficacy positively influence online instructional effectiveness, while Sun and Mei (2022) showed that preservice teachers with higher technology-related self-efficacy developed more favorable attitudes toward digital tools, which in turn strengthened their intention to integrate technology. Similarly, Yang and Lou (2024) reported that teachers with greater digital literacy self-efficacy were more likely to adopt educational technologies, and Liu (2020) confirmed that robust technological support during training enhances TPACK self-efficacy, which predicts actual technology integration in classrooms. Career development and retention also fall within internal domains. Sun (2021) demonstrated that teaching assistants with stronger self-efficacy displayed greater initiative and stronger career intentions, while Sun et al. (2023) revealed that very low self-efficacy undermined professional commitment. Notably, excessively high self-efficacy, when combined with unmet expectations, may also pose challenges, whereas balanced efficacy and realistic optimism reinforced long-term engagement. Teacher self-efficacy further influences emotional and psychological resources. Chan (2008) found that although self-efficacy alone did not predict coping strategies, in combination with emotional intelligence it shaped teachers’ ability to manage stress, underscoring its role in psychological resilience.

External domains highlight how teacher self-efficacy interacts with social and institutional contexts. Bao et al. (2016) showed that encouragement and positive feedback from colleagues and family were critical in rebuilding a teacher’s self-efficacy after traumatic experiences, demonstrating the importance of social persuasion in sustaining resilience. Zhu and Li (2023) extended this perspective by analyzing collective resilience, showing that teachers with higher self-efficacy actively engaged in institutional collaboration, which enhanced both individual adaptability and organizational growth.

Overall, these findings indicate that TCFL teacher self-efficacy exerts predictive effects on multiple variables across both internal and external domains. Internally, it shapes technology use, career commitment, and emotional resilience, while externally, it operates through social support and institutional dynamics to foster adaptability and professional sustainability, as illustrated in Figure 2.

## 5 Discussion

This systematic review revealed several important findings regarding the self-efficacy of TCFL teachers. First, the research topics addressed in the literature are diverse, ranging from the antecedents of self-efficacy (personal, student, and environmental factors) to the outcomes associated with self-efficacy (such as Technology use and integration, Career development and retention as well as Emotional and psychological resources). This diversity shows that TCFL teacher self-efficacy has been studied both as something influenced by context and as something that influences teachers’ behaviors and attitudes. Second, many studies in this area employ qualitative or mixed-methods designs, reflecting an effort to capture the context-specific nuances of TCFL teacher self-efficacy, a trend that aligns with calls in the broader literature for more in depth, contextualized investigations of teacher efficacy (Wyatt, 2018). Moreover, a noticeable gap remains: none of the reviewed studies directly examined the impact of TCFL teacher self-efficacy on student learning outcomes, mirroring Hoang (2018) observations in EFL settings that the connection between teacher self-efficacy and student achievement is often assumed rather than empirically tested.

Many of our findings corroborate the patterns reported in the literature on general teacher self-efficacy. The influential factors identified, such as teaching experience, support from school leadership, and targeted professional development, echo the conclusions of prior reviews in mainstream education contexts (Klassen et al., 2011; Wray et al., 2022) that emphasize the importance of environmental and personal resources in shaping teachers’ efficacy beliefs. Similarly, the positive relationships observed in TCFL contexts between teacher self-efficacy and their willingness to adopt new instructional strategies or technologies are consistent with existing evidence that teachers with higher self-efficacy tend to be more effective, innovative, and persistent in teaching (Cantrell et al., 2013; Mojavezi and Tamiz, 2012). This review highlights several unique aspects of the TCFL context. For example, several studies (e.g., Sun et al., 2023) have highlighted the challenges of teaching Chinese in non-native environments and how factors such as cross-cultural adaptation and language barriers can moderate the role of self-efficacy, which is less pronounced for teachers of more commonly taught languages. These nuances underscore Bandura’s (1994) assertion that self-efficacy is context dependent and validate the need for domain-specific reviews, specifically addressing the TCFL context, thereby responding to calls for more localized analyses of teacher self-efficacy (Klassen et al., 2011; Wyatt, 2018).

The results of this review suggest that enhancing TCFL teacher self-efficacy requires a more comprehensive approach. It is not enough to focus only on improving individual teachers’ skills or providing external support; both personal competency-building and supportive contextual conditions are necessary. For example, opportunities for mastery experiences through well-designed professional development coupled with positive vicarious experiences (such as mentoring by successful peers) and strong organizational backing are likely to bolster teachers’ confidence (Bandura, 1997). Strengthening self-efficacy in these ways is expected to yield multiple benefits: more confident TCFL teachers are likely to implement effective teaching practices, show greater resilience in the face of challenges, and remain committed to the profession, all of which can contribute to better student engagement and learning outcomes in Chinese-language classrooms. In summary, our review provides timely consolidation of research in an emerging field and offers insights that can inform both theoretical frameworks (by validating the applicability of self-efficacy theory in the TCFL domain) and practical efforts to support Chinese-language teachers.

Building on the synthesized literature and its theoretical implications, the following sections explore two key dimensions: how stakeholders can act to support TCFL teachers in practice and how researchers can further investigate unresolved questions in this domain. Together, these findings provide a comprehensive outlook for advancing both policy and theory in the field.

### 5.1 Recommendations for practice

To increase the self-efficacy of TCFL teachers, measures need to be taken at both the organizational and individual levels.

First, from an organizational perspective, strengthening teacher training and PD is crucial. Research indicates that continuous professional learning opportunities, particularly for novice teachers, help build confidence in their teaching practices (Zhang et al., 2020). Encouraging critical reflection allows teachers to evaluate and adjust their teaching methods (Polin, 2023). Additionally, facilitating communication and collaboration among teachers fosters a supportive professional community, which is essential for enhancing professional satisfaction and self-efficacy (Polin, 2021, 2023). As Zhu and Li (2023) suggested, self-efficacy is a vital resource that supports resilience and professional growth.

Therefore, improving the teaching environment and conditions is essential. Considering the impact of environmental factors on self-efficacy, Chen and Yeung (2015) suggest that schools should aim to create a positive working atmosphere and provide adequate resources and support. In online teaching contexts, the availability of technological facilities is crucial for ensuring that teachers have the necessary tools to facilitate effective pedagogical interactions (Bao et al., 2021). Lin and Zheng (2015) emphasized the significant influence of pedagogical and technological self-efficacy on online language teaching practices.

School management should recognize the importance of teachers’ professional well-being by providing emotional support and recognition, helping them feel valued in their work (Bao et al., 2022). Additionally, positive social persuasion, such as support from family and peers, can significantly strengthen teacher self-efficacy during pivotal moments (Bao et al., 2016).

Adapting to multicultural environments is vital for TCFL teachers. Schools should provide intercultural communication training to help teachers effectively navigate linguistic and sociocultural differences effectively (Sun et al., 2023). In non-target-language environments, teachers may require additional support to address challenges and ensure effective teaching. Sun (2021) highlighted that self-efficacy is crucial for the career planning of TCFL teaching assistants, with those with relevant backgrounds showing greater confidence in their teaching roles.

In this context, financial support and incentives are important considerations. Providing adequate financial support can alleviate the financial pressures that teachers face (Liu and Sayer, 2016). Schools should implement reasonable enrollment plans and communicate goals with teachers to address challenges that may arise during the enrollment process collaboratively. Moreover, strengthening technology self-efficacy has been shown to positively impact teachers’ attitudes toward technology use, thereby enhancing their ability to integrate educational technology into the classroom (Sun and Mei, 2022).

Second, from an individual perspective, focusing on student factors is equally important. Building strong teacher–student relationships and fostering student motivation and engagement not only enhances teacher self-efficacy but also leads to better teaching and learning outcomes (Chen and Yeung, 2015). Schools and teachers should develop strategies that address student motivation and classroom discipline, cater to the diverse needs of students and stimulate their interest in learning.

The evaluation of emotional feedback and support is critical. Encouraging students to provide positive affective feedback, such as trust and support, is essential for enhancing their self-efficacy (Bao et al., 2021).

### 5.2 Future research directions

While existing research has provided valuable insights into the self-efficacy of TCFL teachers, several areas remain underexplored and warrant further investigation.

First, in terms of factors influencing self-efficacy, although existing studies (e.g., Chen and Yeung, 2015) have highlighted key factors such as language proficiency, professional development, and teaching experience, future research should further explore how these factors vary across different teaching contexts. Additionally, investigating the recovery process of teachers after encountering challenges, as Bao et al. (2016) suggested, could reveal how positive social persuasion rebuilds confidence, offering insights into resilience-building in diverse settings. Understanding these influences in various settings will allow targeted interventions to support teacher resilience and confidence.

Second, with respect to self-efficacy and other variables, with technology playing an increasing role in education, future research should focus on how specific technologies impact TCFL teacher self-efficacy and determine the best training practices for technology integration. Lin and Zheng (2015) and Sun and Mei (2022) have shown that technological self-efficacy influences teaching effectiveness, especially in online environments. Yang and Lou (2024) also highlighted the importance of digital self-efficacy in teachers’ technology acceptance. As educational technology evolves, examining advanced tools such as artificial intelligence and virtual reality may provide new insights into how TCFL teachers adapt to digital classrooms.

Third, the relationship between career development and self-efficacy warrants further attention. While Sun (2021) explored this link among junior teachers, future research could examine self-efficacy across all career stages to better understand its role in professional growth and job satisfaction. Expanding on Polin’s (2023) work, designing effective career development pathways and institutional incentives may be essential for sustaining high self-efficacy throughout a teacher’s professional trajectory.

Fourth, emotional and psychological resources should be further examined. While a few studies (e.g., Chan, 2008; Zhu and Li, 2023) have explored how teacher self-efficacy interacts with coping strategies, resilience, and collective well-being, this area remains underexplored in TCFL contexts. Future research could investigate how efficacy beliefs shape teachers’ ability to manage stress, maintain motivation, and contribute to collective resilience, thereby offering a fuller understanding of the psychological mechanisms that sustain effective teaching.

Fifth, given the challenges faced by TCFL teachers in multicultural environments. Sun et al. (2023) suggested that future studies should investigate how cross-cultural adaptation influences self-efficacy. Research on developing culturally responsive teaching strategies and training programs can enhance teachers’ adaptability, thus benefiting their self-efficacy and teaching effectiveness in diverse settings. The link between teacher self-efficacy and students’ learning outcomes has been explored in general educational contexts, but more empirical research is needed, specifically for TCFL. Understanding how enhanced teacher self-efficacy improves student motivation, achievement, and classroom management in TCFL settings can guide the development of effective instructional strategies (Mojavezi and Tamiz, 2012).

Finally, in terms of methodological approaches, future research should employ a variety of methodological approaches to provide a more comprehensive understanding of teacher self-efficacy. Triangulation should be pursued not only across methods but also across data sources, by combining surveys, interviews, classroom observations, and document analysis, thereby strengthening the robustness of evidence. Mixed method designs, as recommended by Zhu and Li (2023), can offer a comprehensive approach to understanding self-efficacy in TCFL. Creswell and Plano Clark (2018) argue that mixed-methods research compensates for the weaknesses of both quantitative and qualitative research. On the one hand, quantitative research cannot provide a full picture of people’s living environments and does not allow participants to share their experiences and perspectives. However, qualitative research may involve personal interpretation and bias by the researcher, and the results may not be generalizable due to the limited number of participants. In the study of teacher self-efficacy beliefs, a mixed methods design can triangulate survey and interview data. Additionally, the integration of both sets of data can help researchers better understand the rich descriptions in teacher narratives, which is valuable for teacher educators (Polin, 2021; Wyatt, 2013, 2014). By integrating quantitative methods (e.g., surveys and SEM) with qualitative approaches (e.g., interviews and narrative analysis), researchers can generate richer and more reliable insights into the formation and development of self-efficacy in TCFL contexts.

## 6 Conclusion and limitations

This study provides a comprehensive review of existing research on teacher self-efficacy in the field of TCFL. In response to the four guiding questions, several key insights have emerged. First, by analyzing the included studies, we identified critical research themes and findings (Q1), particularly the factors influencing TCFL teacher self-efficacy, including professional development, supportive teaching environments, student engagement, emotional and social support, cultural adaptation, and financial incentives (Q3). Second, the review showed that methodologically, most studies relied on qualitative and self-reported designs, supplemented by a smaller number of quantitative and mixed methods approaches (Q2). This reflects the context-dependent nature of self-efficacy research but also highlights the need for more diversified methodologies. Third, our synthesis indicates that teacher self-efficacy predicts important outcome variables (Q4), such as technology use and integration, career development and professional retention, emotional resilience, job satisfaction, and overall instructional quality, all of which are essential to effective TCFL practice. Recommendations for practice highlight the need for continuous professional development, supportive infrastructure for both in-person and online teaching, and cross-cultural training tailored to the unique challenges faced by TCFL teachers. These strategies not only enhance self-efficacy but also contribute to sustainable career growth and improve teaching effectiveness. Furthermore, future research directions suggest exploring the impact of technology, career development across teaching stages, and cross-cultural aspects of self-efficacy to expand our understanding and support TCFL educators more effectively.

By consolidating insights from multiple studies, this review lays a strong foundation for advancing teacher self-efficacy research within the TCFL domain. These insights have practical implications for educational policymakers, school administrators, and teacher training programs, offering guidance for fostering an environment that promotes teachers’ confidence, adaptability, and professional growth. Ultimately, enhancing TCFL teacher self-efficacy will not only enrich their teaching experiences but also positively impact student learning outcomes and the broader goals of international Chinese language education.

This study also has some limitations. As with many systematic reviews in education and applied linguistics, the restriction to English-language publications may have led to the exclusion of relevant research in other languages, thereby limiting contextual diversity. Additionally, the number of eligible empirical studies remains relatively small, and the dominance of qualitative and self-reported data constrains the generalizability and causal inference of the findings. Nevertheless, by following the PRISMA framework and adopting a multi-database search strategy while also including rigorously evaluated doctoral dissertations, this review sought to maximize the available evidence and ensure methodological transparency. Although these limitations are characteristic of reviews in developing research areas, the present study has systematically synthesized current knowledge to provide a meaningful and reliable outlook on TCFL teacher self-efficacy.

## Data Availability

The original contributions presented in the study are included in the article/supplementary material, further inquiries can be directed to the corresponding author.
