# Correction: Progress or plateau? A 20-year systematic review of self-efficacy among TCFL teachers

**DOI:** 10.3389/fpsyg.2025.1745343

**Published:** 2025-11-24

**Authors:** Qian Shi, Jing Zhang, Wei Zhao

**Affiliations:** 1 School of Chinese Language and Literature, Yunnan University, Kunming, China; 2 School of Entrepreneurship, Zhejiang University of Finance and Economics Dongfang College, Haining, China

**Keywords:** teacher self-efficacy, teaching Chinese as a foreign language (TCFL), social cognitive theory, professional development, cross-cultural adaptation

**Author affiliation**

Affiliation 2 was erroneously written as:

Dongfang College, Zhejiang University of Finance and Economics, Haining, China

The correct affiliation is:

School of Entrepreneurship, Zhejiang University of Finance and Economics Dongfang College, Haining, China

The original version of this article has been updated.

